# Experimental study on strengthening interior beam column joints in reinforced concrete structures using wing walls

**DOI:** 10.1038/s41598-025-34376-7

**Published:** 2025-12-30

**Authors:** Li Yuebing, Yan Qi, Wang Hang, Han Xitian, Xing Shuang

**Affiliations:** 1 School of Civil Engineering and Architecture, Northeast Electric Power University, Jilin, 132012 China; 2 CGN Engineering Co, Shenzhen, 518000 China; 3 Zhengzhou Branch, Huadian Heavy Industries Co., Ltd, Zhengzhou, 450009 China; 4 State Grid Changchun Electric Power Supply Company, Changchun, 130012 China

**Keywords:** Reinforced concrete, Interior beam‒column joint, Wing wall installation, Seismic strengthening, Designing method, Engineering, Materials science

## Abstract

Early damage to beam‒column joints during an earthquake can cause rapid degradation of structural seismic performance or even collapse of buildings. Installing reinforced concrete (RC) wing walls to one side of the columns of an existing RC frame structure, i.e., enlarging the column cross section, can not only improve the strength of the columns but also strengthen the beam–column joints. Quasistatic cyclic loading tests were conducted to validate the strengthening effectiveness for interior beam‒column joints by wing wall installation methods, and the strengthening mechanism was analyzed. Through theoretical analysis, a mechanical model and a simplified design method for strengthening interior beam-column joints using wing walls were proposed. The method focuses specifically on the contribution of the compressive wing wall to the joint strengthening effect. The test results revealed that for the non-strengthened benchmark specimen, damage was concentrated at the joint region. The strength of the strengthened specimen was largely improved, and an ideal beam-yielding mechanism was formed. A mechanical model and design theory for strengthening interior beam‒column joints was proposed, with which the maximum strengths and failure modes of the specimens with/without strengthening were evaluated. This study reveals that seismically deficient interior beam-column joints, which are subjected to shear forces up to twice those of exterior joints during seismic events, can be effectively strengthened using a wing wall installation method. A novel, simplified design approach is proposed for this strengthening technique, offering improved practicality and efficiency compared to existing methods.

## Introduction

 Recent major earthquake disasters have shown that the seismic capacity of urban and rural buildings is still relatively weak^1–5^. Currently, China’s urban construction is in the stage of shifting from mainly being newly built to the coexistence of newly built and existing improvements^6^. The collapse of buildings is the main cause of casualties in earthquakes, and improving the collapse resistance of existing buildings is the main method for mitigating seismic disasters^7^.

Beam‒column joints are important force transfer hubs in reinforced concrete (RC) frame structures, and the Chinese seismic code clearly requires a building be designed with"strong joints and weak members“^8^. This design ensures that the beam-column joints are stronger than the adjoining beams and columns. Hence, when analyzing the seismic performance of a frame structure, the joints are usually assumed to be rigid. However, seismic disasters in recent years have resulted in a large amount of damage being observed in RC structures, which have been considered to have excellent seismic performance^9^. One of the damage characteristics is joint failure, which fails to achieve the design objective of rigid joints^10^. Joint failure leads to a rapid increase in the horizontal displacement of the frame structure under seismic action, leading to a story or complete collapse mechanism under the additional moments caused by vertical loads.

Researchers have conducted extensive investigations into the structural assessment of RC structures. Gong^11^ proposed a numerical simulation strategy based on the rigid body spring model to predict the fracture behavior of RC structures, which was validated through tests on a simply-supported RC beam. Yang^12^ reviewed the performance requirements of 3D concrete printing for printing materials, examined existing methods for characterizing material printability, and suggested directions for material optimization. Li^13^ developed a multi-criteria benefit evaluation framework for recycled carbon fiber (RCF)-reinforced mortar using a hybrid weighting methodology. Zhai^14^ introduced an optimal design procedure for damped structures under multi-level seismic actions. Long^15^ established a finite element macromodel to reliably simulate the progressive collapse resistance of both seismic and non-seismic RC beam-column sub-assemblages. Innovative structural systems have also been proposed, such as the steel-hollow partially encased composite spliced frame beam system^16^. Zhang^17^ developed a hybrid reinforced concrete beam that combines steel and fiber-reinforced polymer (FRP) rebars to enhance durability while maintaining load-bearing capacity. Regarding strengthening techniques for seismically deficient structures, various methods have been explored, including cable bracing^18^, FRP sheet bonding^19^, and the use of high-performance ferrocement laminate (HPFL)-bonded steel plates^20^.

However, for weak RC beam‒column joints, it is difficult to strengthen the joint zone directly due to the existence of slabs and orthogonal beams. Moreover, the joints are usually considered to be rigid without damage; therefore, strengthening methods for RC joints were not provided in the seismic design code for strengthening concrete structures^21^. The development of an effective strengthening method for RC beam‒column joints with good cost performance is urgently needed.

Although the design code for concrete structures^22^ provides strict structural requirements on reinforcement at joint regions, shear reinforcement is not always arranged in the joint regions due to the lack of professional knowledge or carelessness of the construction workers, especially in developing countries, which is more general due to the lack of effective construction supervision and management for rural buildings^23^. Moreover, reinforcement in the joint region is complex, and the assembly order cannot be reversed.After the assembly is complete, even if the joints are not equipped or are less equipped with shear reinforcement, it is difficult to reassemble. Investigations on earthquake-damaged buildings^3,24^, surveys on structures under construction^23^, and nondestructive tests on existing structures have revealed that insufficient or even no shear reinforcement in joints is common.

Researchers have developed various techniques for seismic strengthening of beam-column joints, including the application of CFRP sheets^25^, brace devices^26^, potassium-activated geopolymer concrete^27^. However, these methods present certain limitations, such as high cost, insufficient fire resistance, limited durability, and the inadequacy of strengthening materials. Consequently, the widespread adoption of these techniques remains challenging, particularly in developing countries where economic and technological resources are constrained. Furthermore, strengthening only the beam-column joints may lead to an alternative undesirable failure mechanism, such as column bending failure—especially in structures with slender columns, a common feature in many developing regions. Moreover, the majority of strengthening tests have been conducted on exterior joints^28–32^, while interior joints have received considerably less attention.

**Fig. 1:**
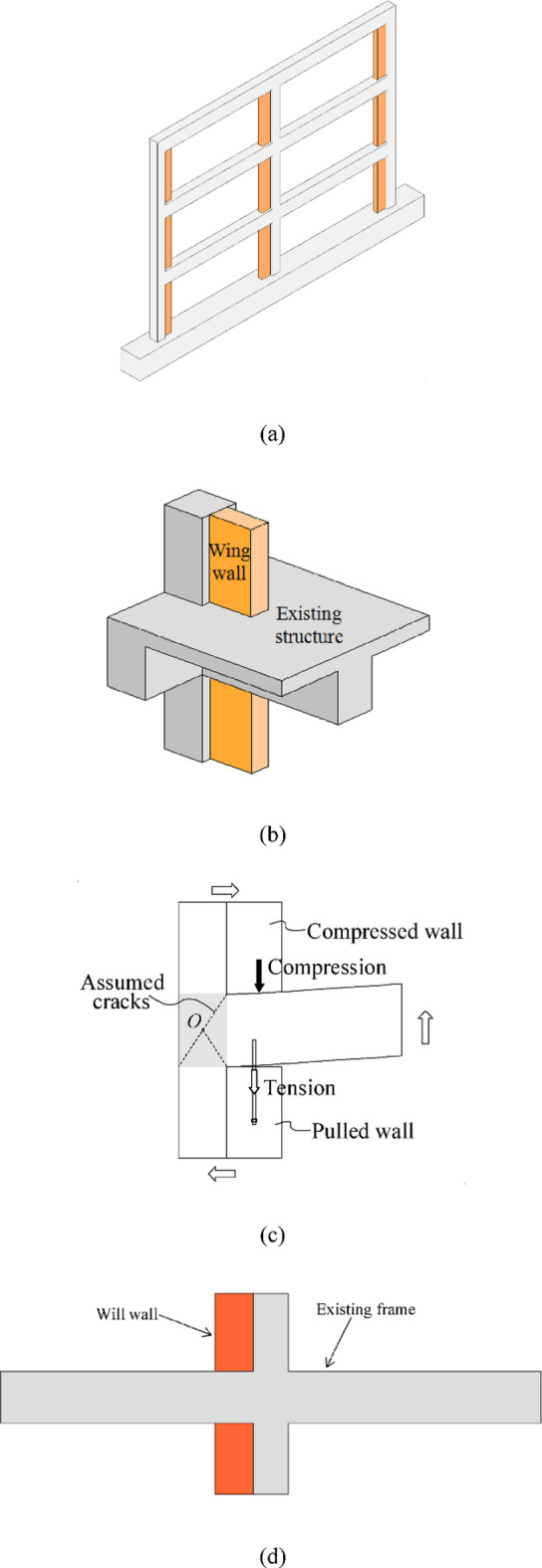
Image of strengthening the RC frame and beam‒column joints by the RC wing wall installation method. (**a**) Strengthening frame. (**b**) Strengthening exterior joint in previous study^33^, (**c**) Strengthening mechanism by wing wall installation^33^, (**d**) Strengthening interior joint in the current study.

Considering the economic and technological limitations in developing countries, LI et al.^33^ proposed strengthening beam-column joints by installing RC wing walls on one side of the columns, as illustrated in Fig. 1(a). The strengthening effect was validated through tests on exterior beam‒column joint specimens, as shown in Fig. 1(b). However, the efficacy of this strengthening technique has not been validated for interior beam-column joints. Such joints are particularly vulnerable, as they experience nearly twice the shear force as exterior joints during seismic activity. Although a strengthening mechanism was proposed in previous study^33^, as depicted in Fig. 1(c), that employs both compressive and tensile forces from post-installed wing walls to improve the yield strength of the joint, assessing the combined contribution of these forces remains challenging in practical engineering design.

In this paper, two specimens were manufactured to represent extremely vulnerable interior beam‒column joints where no shear reinforcement was applied. One of them is strengthened by installing RC wing walls, as shown in Fig. 1(c). Through loading tests, the seismic performance of the joint with no shear reinforcement is studied, and the strengthening effectiveness is confirmed. Moreover, the strengthening mechanism is analyzed. A strengthening mechanical model is proposed, and taking the formation of a beam yielding mechanism as the strengthening objective, a strengthening design method is proposed based on theoretical analysis, which can be used to quantitatively determine strengthening parameters such as wing wall dimensions. Experimental observations revealed distinct brittle failure characteristics in interior beam-column joints with non-compliant reinforcement details. The wing wall installation method was demonstrated to be effective in strengthening such vulnerable joints, significantly enhancing their seismic performance. Furthermore, the proposed simplified mechanical model and corresponding design method were validated through favorable comparisons with experimental results.

## Overview of the test

### Specimens

**Fig. 2:**
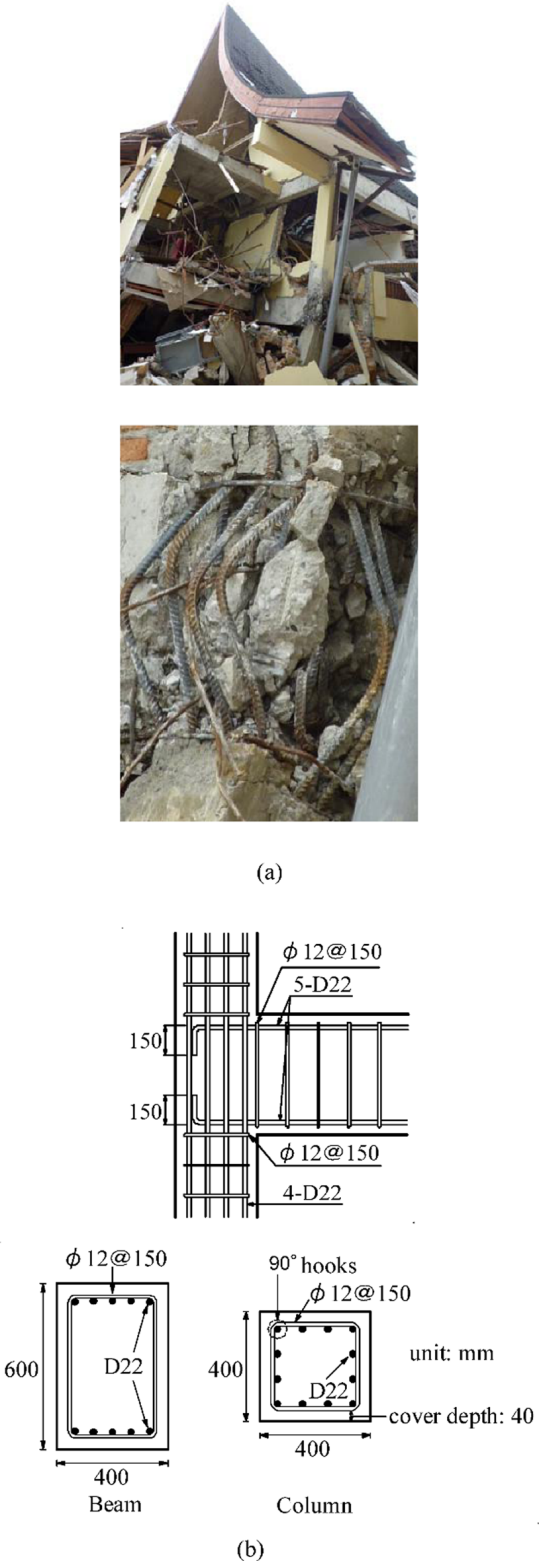
Target building and its damaged exterior beam-column joint. (**a**) View of the building and its damaged beam-column joint^34^, (**b**) Dimensions and reinforcement details of the studied exterior joint^35^.

Based on previous studies^34,35^, a collapsed frame building in the 2009 Sumatra earthquake was taken as the prototype building to design the specimen. The building and details of the beam and column are shown in Fig. 2. In this building, severe damage, e.g., buckling of the column longitudinal reinforcement and core concrete crushing, was observed in the joints. The significant characteristics of the building are that instead of 135° hooks, the hoops in the beams and columns are 90° hooked, and the beam–column joint areas contain no hoops.

Two 1/2-scale specimens were manufactured to model the interior beam‒column joints, one of which was strengthened by installing RC wing walls. The non-strengthened benchmark specimen is named Z1, and the strengthened specimen is Z1-W. However, because of the collapse of the building, the reinforcement details in the interior joints could not be observed; when designing the specimens, the reinforcement in the joints was assumed to be the same as that in the exterior joints^33^. The beams and columns connected to the joints were simulated up to the inflection points (including the size of the connectors to the loading facilities). The strengthening construction was carried out one week after the casting of the existing frame.

**Fig. 3:**
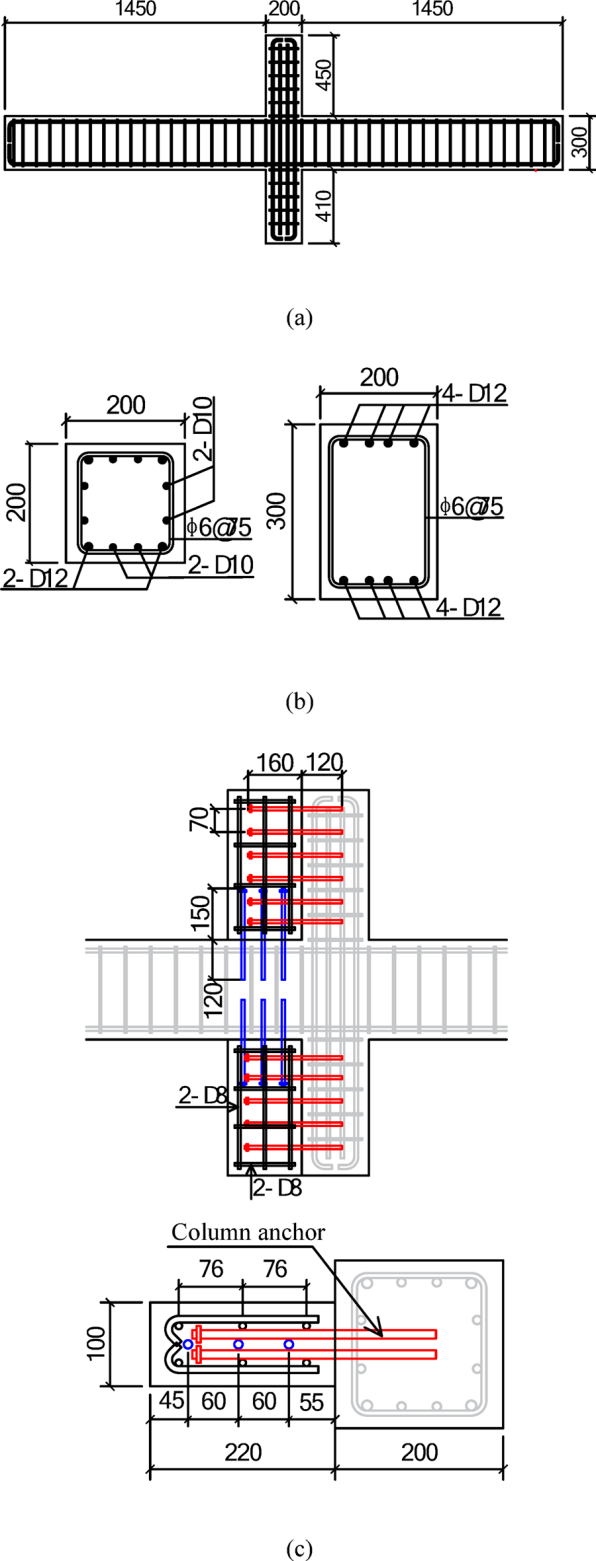
Dimensions and reinforcement details of the specimens. (**a**) Front view of specimen Z1, (**b**) Beam and column section, (**c**) Reinforcement details and dimensions of the wing wall in specimenZ1-W.

The dimensions and reinforcement details of specimen Z1 are shown in Fig. 3(a). Because of the different heights of the upper and lower hinge supports of the columns, the height of the upper column was 40 mm greater than that of the lower column to maintain the same distance from the inflection points to the joint centre.The longitudinal reinforcement consisted of HRB400 grade ribbed steel bars, and the hoops were HPB300 grade plain bars. The design strength of the concrete for the existing frame is C30. As shown in Fig. 3(b), the specimen replicates the structural features of the target building, including the use of 90^o^ hooked hoops in the beams and columns and the absence of shear reinforcement in the joint region. According to mainstream design codes^36–39^, beam-column joints should be provided with adequate shear reinforcement, and stirrups should be bent at 135^o^ to ensure seismic performance. It can be seen that the reinforcement details in the specimen do not comply with these code specifications, thereby categorizing it as a seismically deficient beam-column joint.

**Fig. 4:**
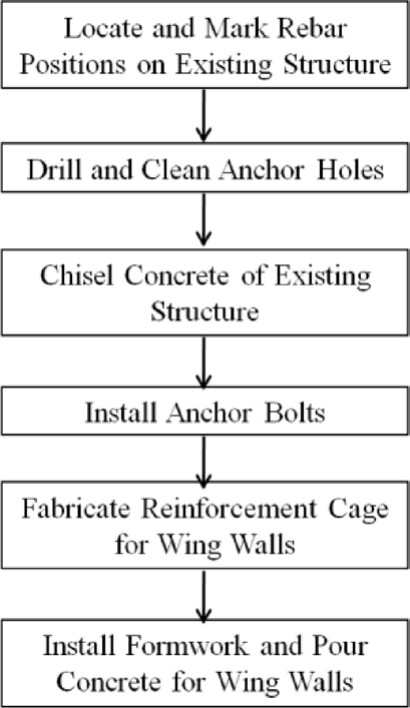
Construction process of wing wall installation.

**Fig. 5:**
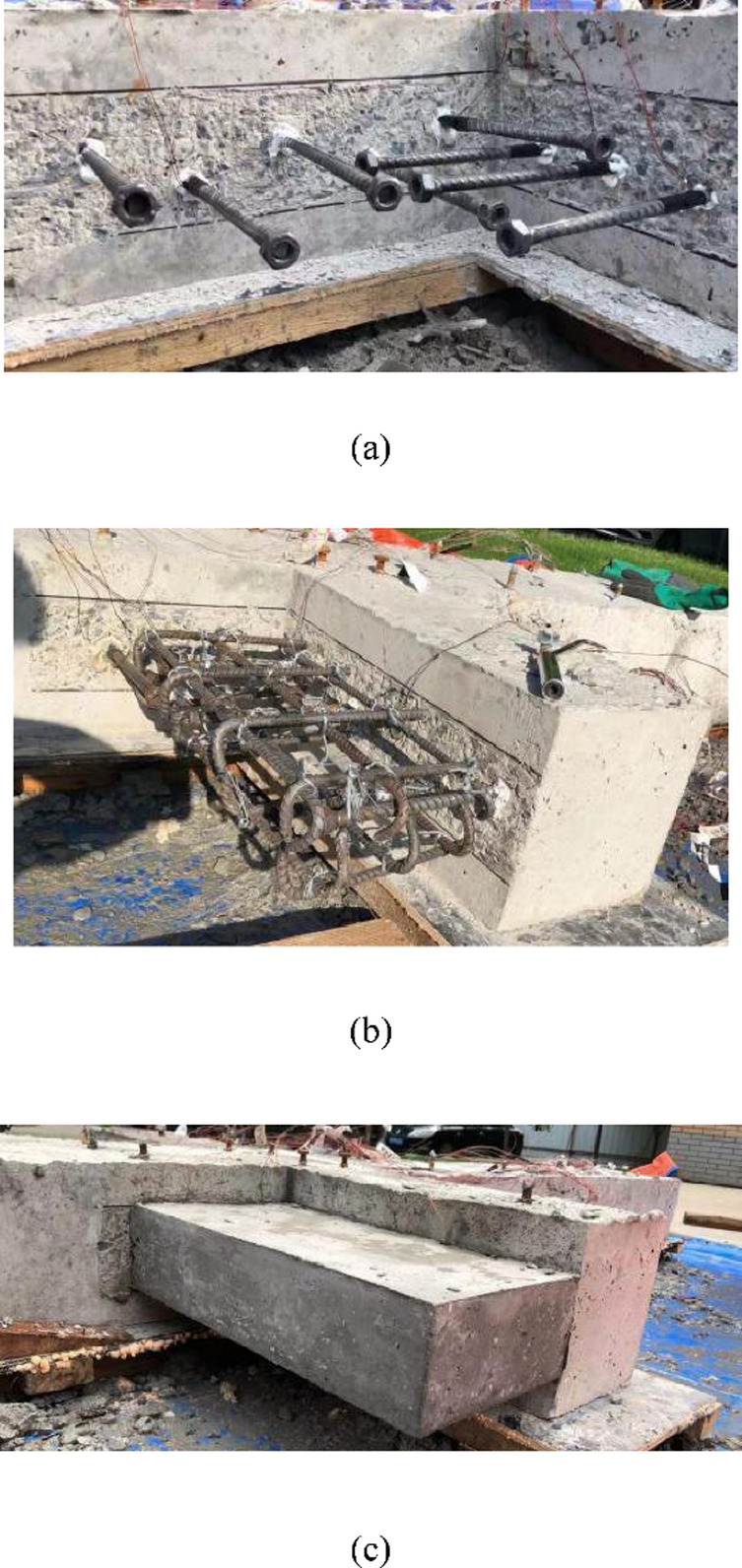
Construction of RC wing walls. (**a**) Post-installed anchors, (**b**) Wing wall reinforcement arrangement, (**c**) Wing wall after removing the formwork.

As shown in Fig. 3(c), the heights of the wing walls in the strengthened specimen Z1-W are the same as those of the columns, and the length and thickness are220 mm and 100 mm, respectively.The design strength of the concrete for the wing walls is C40. The wing walls are connected to the existing frame through post-installed adhesive anchors, which are ribbed steel bars with a diameter of 10 mm with nuts welded to the end at the wing wall side to improve anchoring performance. The construction process of wing wall installation is as as shown in Figs. 4 and 5. The tested concrete compressive strength and mechanical properties of the reinforcement are shown in Tables 1 and 2, respectively.

**Table 1:** Compressive strength of the concrete (MPa).

Design value	Used position	Strength
C30	Existing frame	35.8
C40	Wing walls	46.2

**Table 2:** Mechanical characteristics of the reinforcement (MPa).

Diameter	Yield stress	Tensile strength	Elastic modulus
ϕ8	464.8	684.7	2.12 × 10^5^
D10	478.1	733.2	2.15 × 10^5^
D12	437.5	604.8	2.13 × 10^5^

### Test setup and measurement

**Fig. 6:**
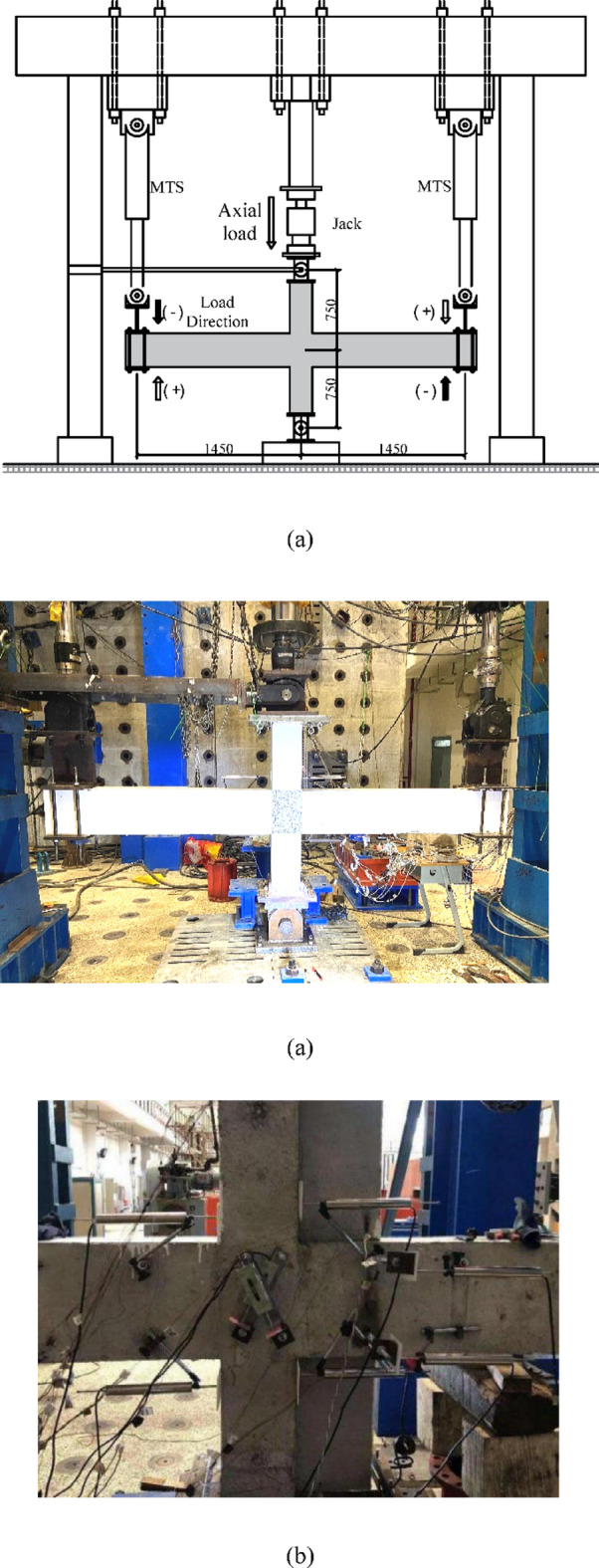
Test setup. (**a**) Loading method. (**b**) Photo of the specimen and loading facilities. (**c**) Displacement meter arrangements.

The test setup is shown in Fig. 6. The upper and lower ends of the columns are fixed to the oil jack for axial force and a hinged pin fixed to the ground, respectively, and the beam ends are free. A constant axial force of 143 kN was applied to the column with an axial force ratio of 0.1. Two vertical 500kN electrohydraulic servo actuators were connected to the beam ends to apply cyclic loads opposite each other. After a constant axial load was applied to the column, quasi-static cyclic displacements were imposed at both beam ends under displacement control. Loading cycles were conducted at drift ratios (*R*) of 0.25%, 0.5%, 1.0%, 1.5%, 2%, 3%, 4%, 5%, 6%, 8%, 9%, and 10%. The direction in which the left beam is pulled upwards and the right beam is pushed downwards is defined as the positive loading direction. The opposite loading direction is defined as the negative loading direction.

During loading, the column axial force, actuator load, beam end displacement, reinforcement strain at critical sections, beam end rotation angle, and joint shear deformation were recorded. The strains of the concrete in the joint region were measured at the peak displacement of every loading cycle using a non contact strain measurement system called VIC-3D.

## Damage process

**Fig. 7:**
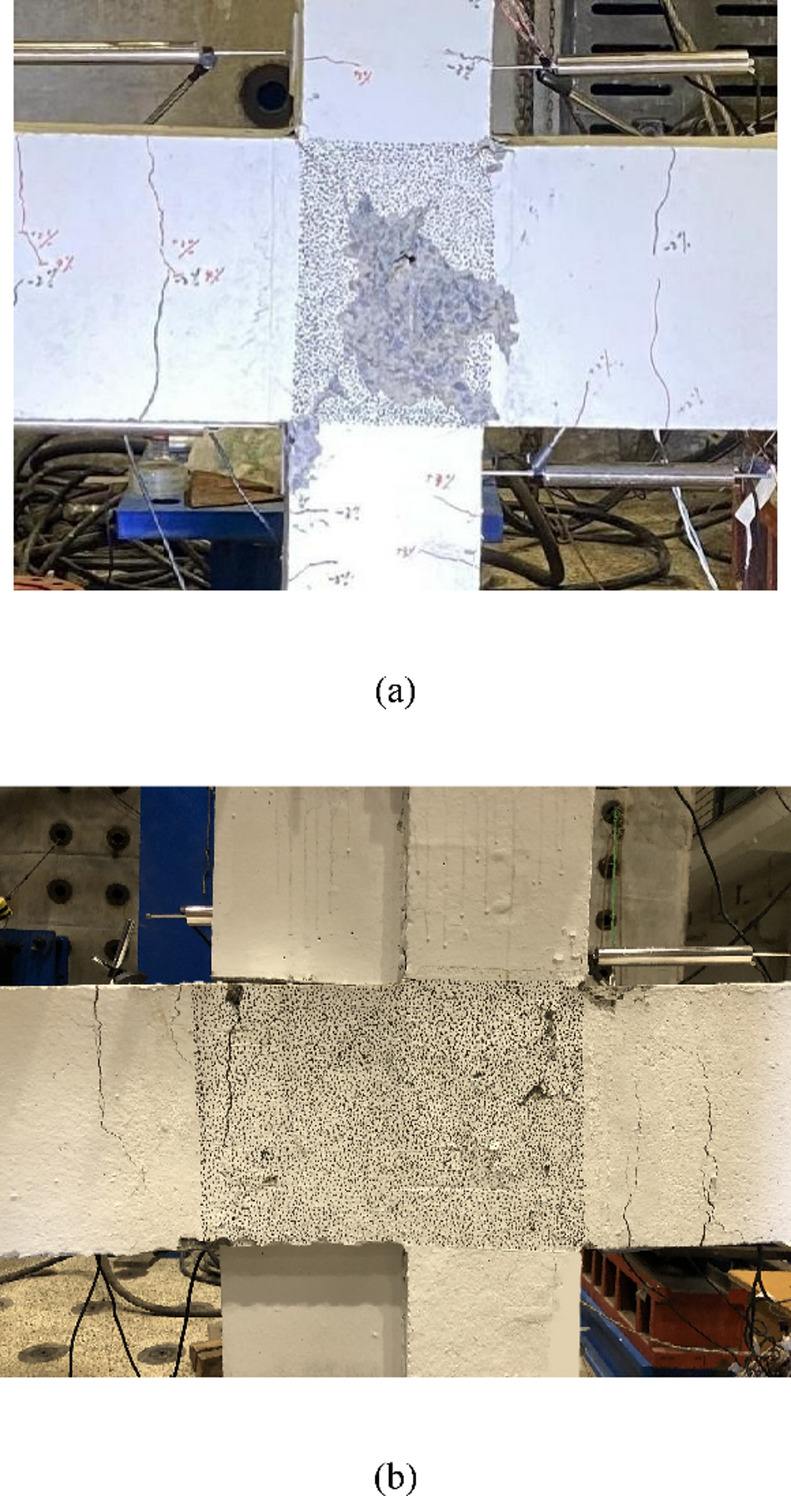
Final damage conditions around the joint region. (**a**) Z1, (**b**) Z1-W.

The non-strengthened benchmark specimen Z1 did not crack until the drift ratio *R* = 0.5%. When loaded to a drift ratio of *R* = 1%, a fine diagonal crack appeared in the joint region in the positive and negative directions, respectively, by forming an X shape.At *R* = 2%, multiple flexural cracks appeared at the critical sections of the beams. When the loading reached *R* = 3%, the diagonal cracks in the joint region increased and gradually expanded, and the cracks in the beams increased; multiple flexural cracks appeared in both the upper and lower columns. When loaded to *R* = 4%, the cracks mainly developed in the joint region, with the beam cracks expanding slightly; vertical cracks appeared at the column top and bottom under axial force.At *R* = 5%, the concrete at the joint region began to peel, and at *R* = 7%, the concrete largely peeled.At *R* = 8%, the concrete in the joint core region was completely crushed, and the reinforcement was exposed. During loading, multiple cracks appeared in the beams and columns, but the expansion was not obvious, and concrete crushing was not observed.The final damage conditions around the joint region of the specimens are shown in Fig. 7, where the black dots in the joint region are used for non contact strain measurements of the concrete. As shown in Fig. 7(a), damage to the benchmark specimen is mainly concentrated in the joint region, indicating obvious shear damage.

For the strengthened specimen Z1-W, no obvious damage was observed until the drift ratio reached 2%.When loaded to + 3%, flexural cracks appeared at the critical section of the beams, and a tiny horizontal crack appeared at the boundary of the left beam and the upper wing wall; fine diagonal cracks appeared at the joint region.At *R* = 5%, the concrete crushed at the critical section of the right beam. During the positive loading direction to *R* = 7%, the bottom of the lower column was split due to the problem of the connector of the specimen. Hence, to ensure that the loading tests were conducted smoothly, the positive loading was stopped, but the negative loading continued. Then, with increasing drift ratio, the cracks at the beam end gradually extended, and the cracks in the joint region slowly increased. From the final damage situation shown in Fig. 7(b), although cracks appeared in the joint region, no concrete crushing or peeling was observed.The cracks obviously expanded at the beam ends, and the concrete was crushed, showing that the strengthened specimen failed during beam yielding. Moreover, by installing a wing wall on the left side of the columns, the critical section of the left beam was moved to the face of the wing wall.

By comparing the damage to the two specimens, the non-strengthened benchmark specimen failed in joint shear, which did not satisfy the provisions in the seismic code of “strong joints and weak members”. By strengthening, although cracks appeared in the joint region, the damage was effectively controlled, and an ideal beam yielding mechanism was formed.

## Analysis of the test results

### Load‒displacement relationships

**Fig. 8:**
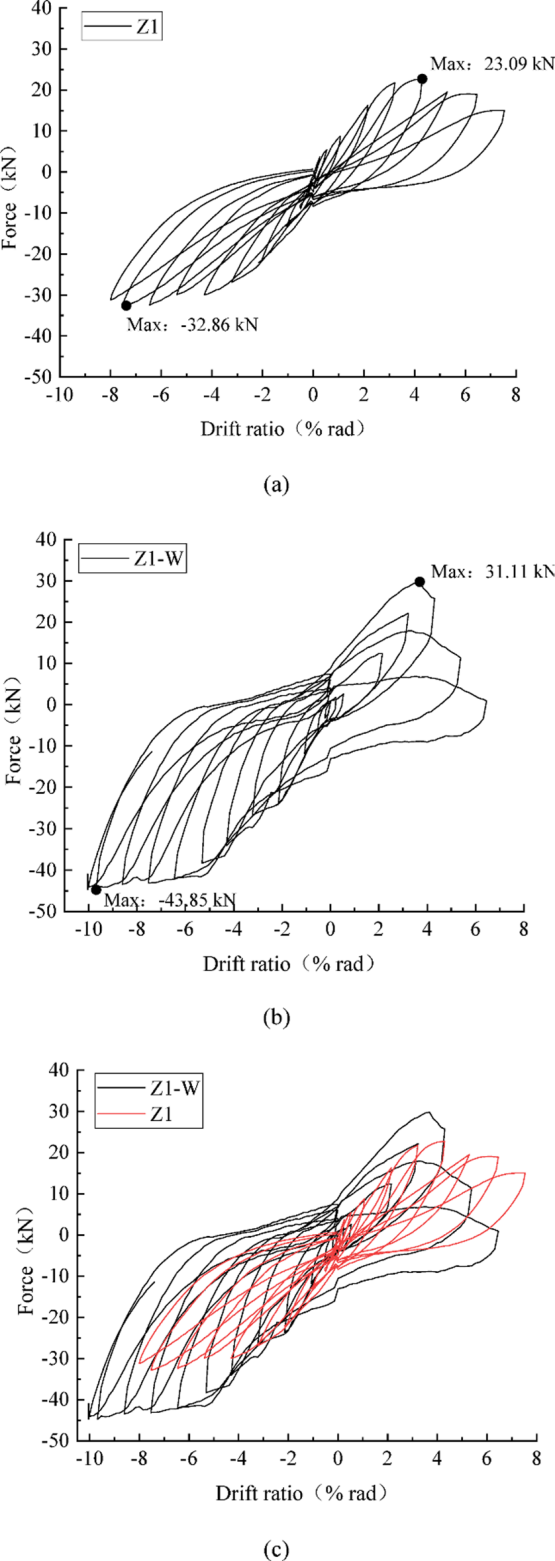
Load‒displacement relationships. (**a**) Z1, (**b**) Z1-W, (**c**) Comparison between the two specimens.

The load‒displacement relationships of the two specimens are shown in Fig. 8. Compared with that of Z1, the strength of the strengthened specimen Z1-W significantly improved. The maximum strength of Z1 was 23.09 kN in the positive loading direction. Because Z1-W was loaded only up to 6% in the positive direction, the hysteresis curve was incomplete, and the maximum strength improved to 31.11 kN under positive loading. Under negative loading, the maximum strength improved by 34%, from 32.86 kN to 43.85 kN.

The deformation ability of Z1-W was also significantly improved. SpecimenZ1 reached the maximum strength at *R* = + 4% and *R*= −7% in the positive and negative loading directions, respectively, followed by a significant decrease in strength. Specimen Z1-W was loaded up to *R*= −10% in the negative direction, and no strength deterioration was confirmed at large deformations.Comparing the pinching phenomenon and energy dissipation capacity of the two specimens, the pinching phenomenon of Z1 is more obvious, and the curves of Z1-W are fuller, indicating that the strengthened specimen has better energy dissipation capacity.

The non-strengthened specimen showed obvious brittle damage characteristics, and by installing wing walls, the strength, deformation capacity, and energy dissipation of the structure significantly improved.

### Stiffness degradation

The stiffness degradation is evaluated by the secant stiffness *K*_i_ by Specification for seismic test of building^40^, which is the ratio of the sum of the absolute values of the positive and negative loads to the sum of the absolute values of the displacements at the peak in each loading cycle, as shown in Eqs. (1),

1 $${K_i}=\frac{{\left| {+{K_i}} \right|+\left| { - {K_i}} \right|}}{{\left| {+{X_i}} \right|+\left| { - {X_i}} \right|}}$$

where, + and - are the loading directions, *F*_i_ is the load at the peak of the *i*th loading cycle, and *X*_i_ is the displacement at the peak of the *i*th loading cycle.

**Fig. 9:**
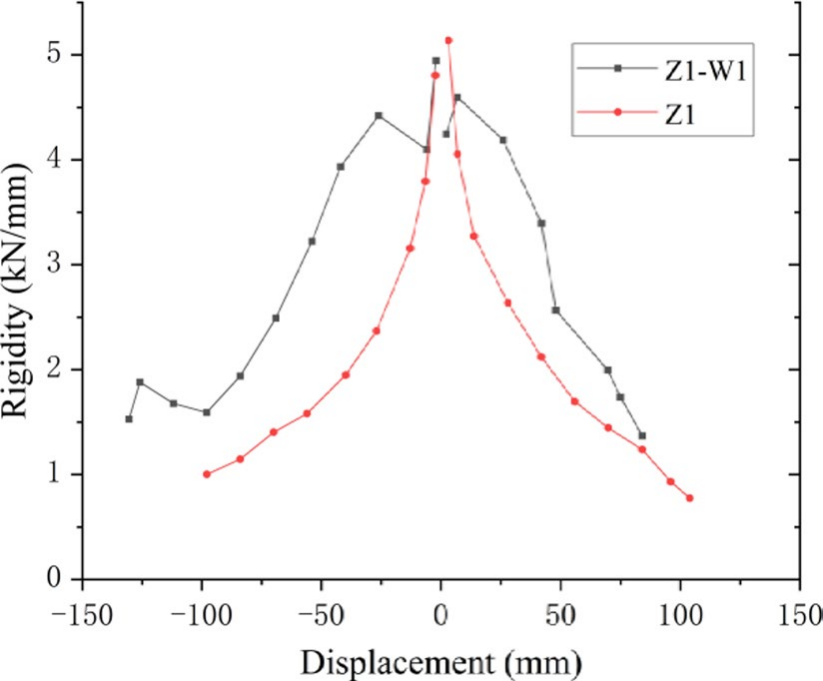
Stiffness degradation.

The results are shown in Fig. 9. The stiffness of specimen Z1-W is always greater than that of specimen Z1, and the stiffness of the structure significantly improved after strengthening.

### Concrete strain at the joint region

**Fig. 10:**
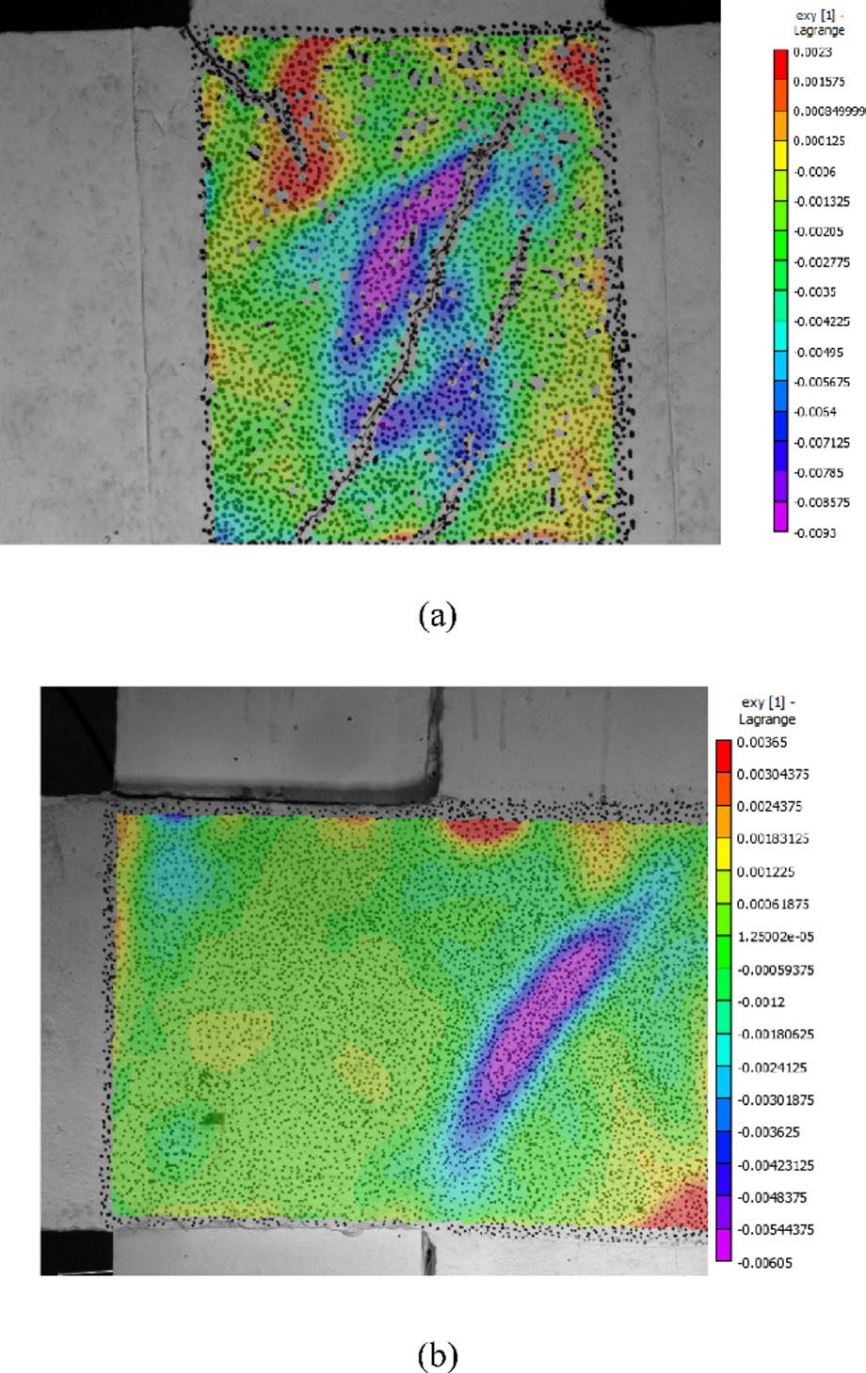
Strain in the concrete at the joint region. (**a**) Z1, *R* = 4%, (**b**) Z1-W, *R* = 4%.

The VIC-3D system was used to observe the strain of the concrete at the joint region, which applied the algorithm by comparing the correlation points of the images, and the displacement and strain results were calculated in real time. Figure 10 shows the strain cloud of the concrete in the joint region at *R* = 4%.

From the figure, it can be seen that an obvious inclined compression strut mechanism was formed in the joint region, and the direction of the inclined compression strut in specimen Z1 was almost along the inclined crack, with a larger tilt angle. The maximum compressive strain was 0.009.An obvious tensile strain appeared at the tensile area of the corner.

The part of the beam where the wing walls are connected and the joint region of the existing structure formed a new joint region, increasing the joint area.The tilt angle of the compression strut is significantly smaller than that of Z1. Before *R* = 4%, the joint region did not exhibit large deformation, and at *R* = 4%, a clear diagonal compressive stress zone appeared, with a maximum strain of 0.006, which was smaller than that of specimen Z1.

### Beam rotation and joint shear deformation

**Fig. 11:**
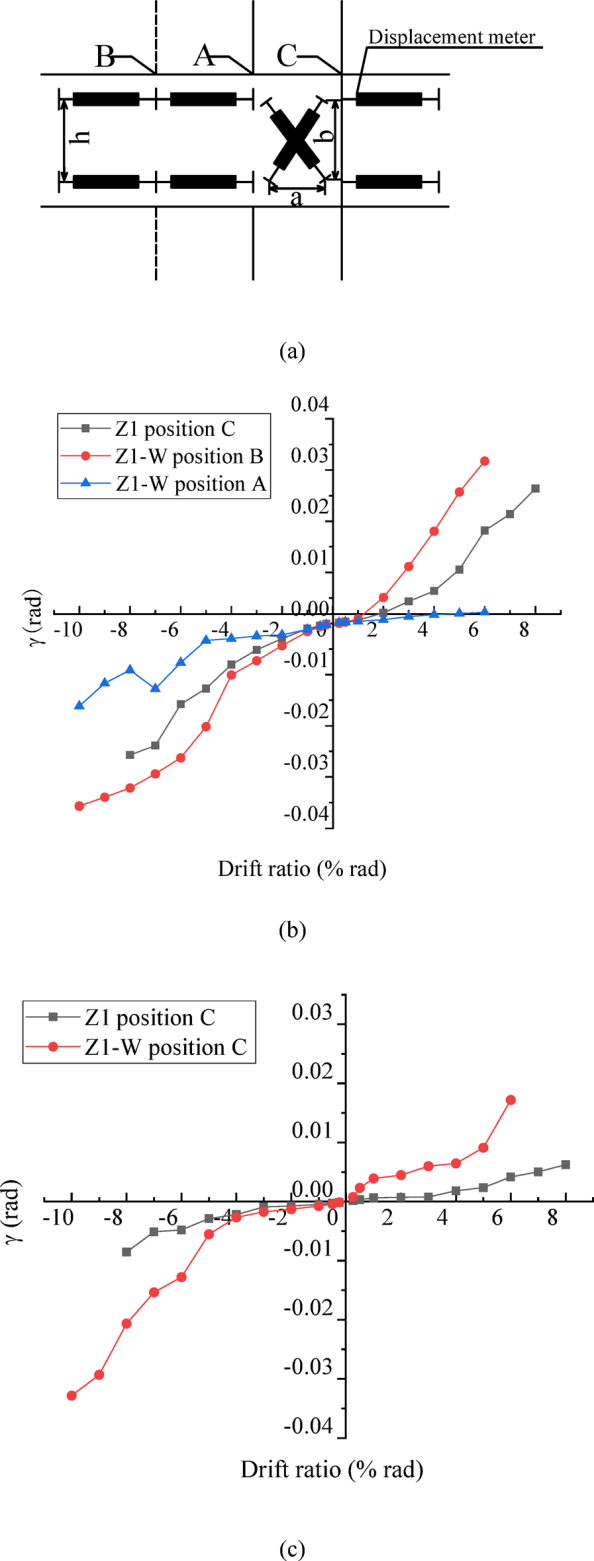
Rotation angle of the beam at the critical section. (**a**) Displacement meter arrangement, (**b**) Critical sections of the left beam where wing walls are installed, (**c**) Critical section of the right beam.

As shown in Figs. 6(c) and 11(a), displacement transducers were arranged horizontally in pairs at the critical section of the beam to determine the rotation angle. Two cross-displacement transducers at the joint region were used to measure the shear drift ratio of the joint. The beam rotation angle with respect to the loading displacement at the peak of each loading cycle is shown in Fig. 11(b) and (c).

As shown in Fig. 11(b), at the left side of the joint, i.e., the beam with the wing wall side, after installing the wing walls, the rotation angle of the beam at location A is smaller than that at location Z1, indicating that the wing walls restrained the deformation of the critical section of the existing structure. The rotation angle of Z1-W at the wing wall face(location B) is much larger than that at location A, indicating that the critical section of the beam shifted from A, and a new critical section formed at the wing wall face of location B after strengthening. The rotation angle at location B of Z1-W is significantly larger than that at location A of the non-strengthened specimen Z1, indicating that the plastic hinge at the beam of the strengthened specimen is more fully developed by forming an obvious plastic hinge.

As shown in Fig. 11(c), the rotation angle of the beam at the right side of the joint, i.e., without wing walls for Z1-W, increased significantly at the critical section (location C) after strengthening, indicating that the plastic hinge at the critical section of the beam without the wing wall was also fully developed after strengthening, which effectively promoted the formation of a beam yielding mechanism.

**Fig. 12:**
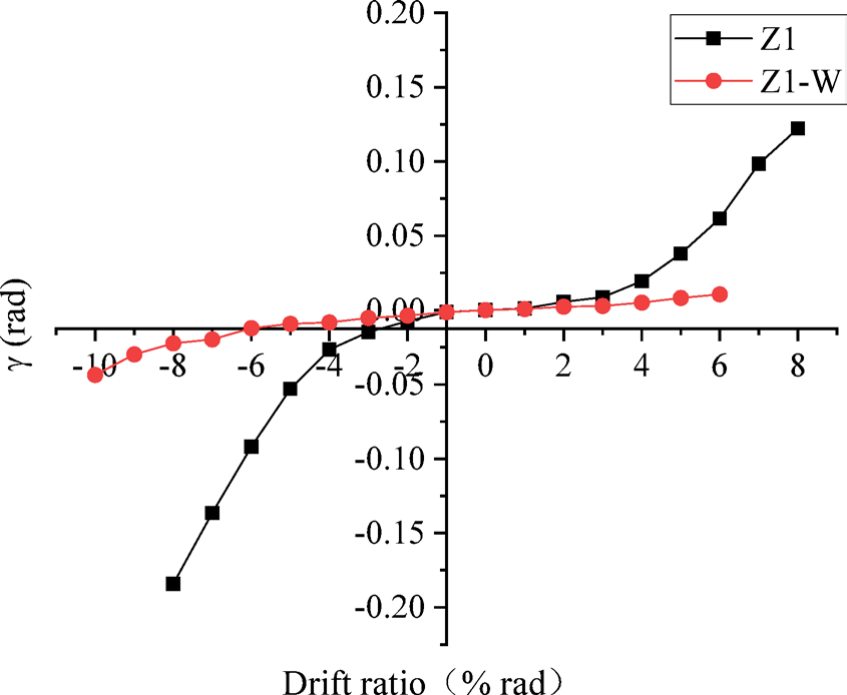
Shear deformation of the joint.

The shear deformation of the joint is shown in Fig. 12. The values of the non-strengthened specimen Z1 were consistently larger than those of the strengthened specimen. The significant shear deformation of the non-strengthened specimen indicates that the behaviour of the joint under seismic action does not agree with the rigid joint assumption in the seismic analysis of a frame structure. The shear deformation of the joint was largely reduced by strengthening, and the overall deformation of the specimen was mainly contributed by beam bending. Although the joint shear deformation cannot be eliminated, it can be reduced to a low level, which is more consistent with the rigid joint assumption in structural seismic analysis.

### Analysis of the reinforcement strain

**Fig. 13:**
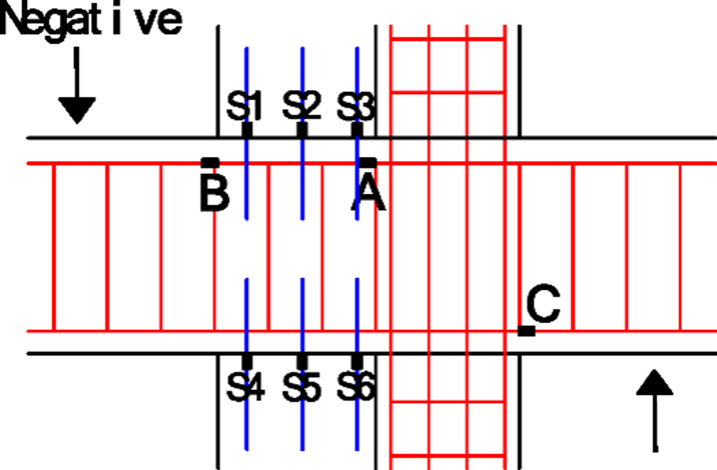
Measured locations and the numbering of reinforcement strains.

**Fig. 14:**
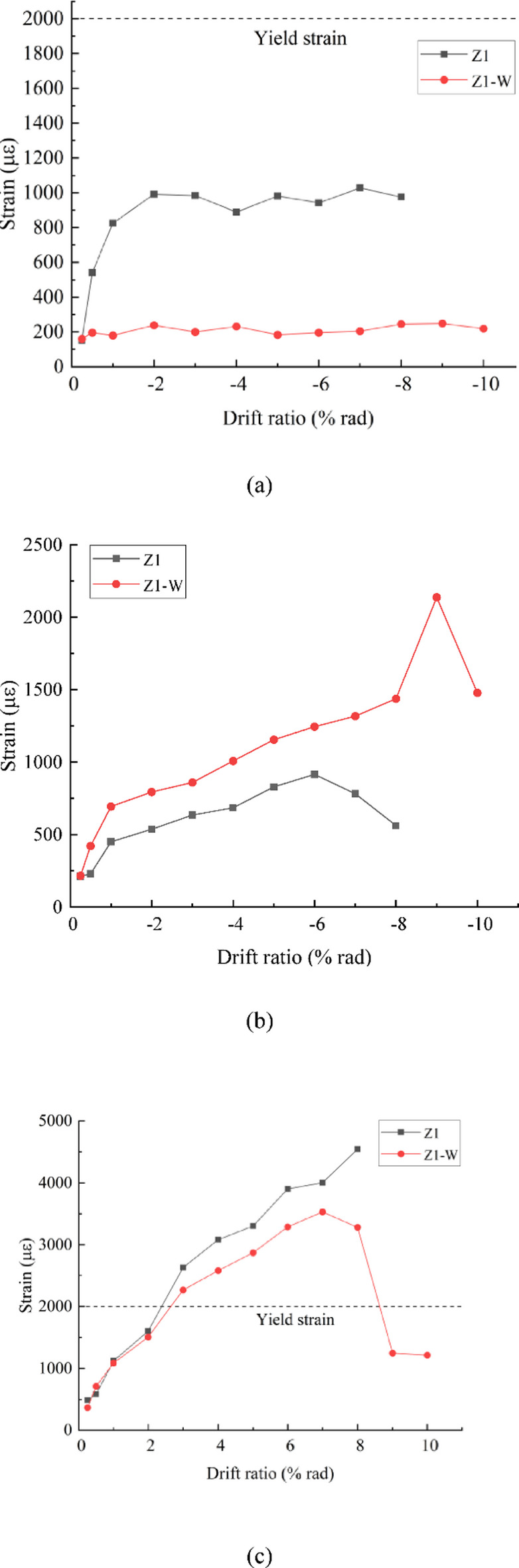
Strains of the longitudinal reinforcement in the beam at the critical section. (**a**) Longitudinal reinforcement in the left beam at the column face of location A in Fig. 10, (**b**) Longitudinal reinforcement in the left beam at location B in Fig. 10, (**c**) Longitudinal reinforcement in the right beam at the column face of location C in Fig. 10.

**Fig. 15:**
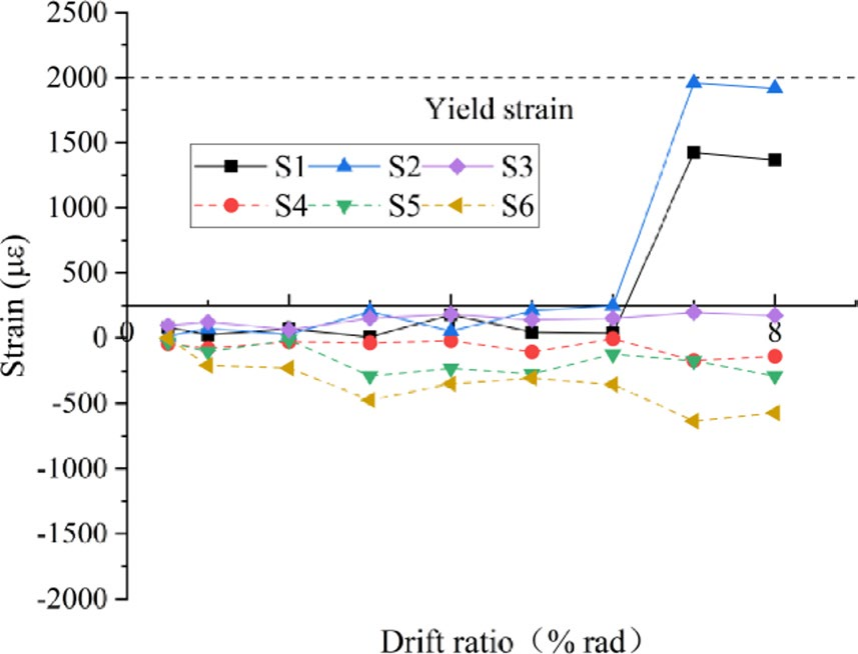
Strains of anchors between walls and the beam.

The location and numbering of the strain gauges for the reinforcement and anchors are shown in Fig. 13. The values of longitudinal reinforcement in the beam and anchor rebars connecting the wing walls and the beam are shown in Figs. 14 and 15, respectively. Because the positive loading to specimen Z1-W stopped earlier during the tests, Figs. 14 and 15 illustrate the results only in the negative loading direction.

#### Strain of the longitudinal reinforcement at the critical sections of the beams

As shown in Fig. 14(a), for the strain of the longitudinal reinforcement in the left beam at the column face (location A), the maximum value of Z1 is approximately 1000 *µ*, which is much smaller than the yield value, indicating that a plastic hinge was not formed at the beam end. The values of the strengthened specimen Z1-W were consistently low, approximately 200 *µ*, indicating that the installation of the wing walls reduced the stress in the longitudinal reinforcement at the beam end of the existing structure and largely reduced the shear force to the joint from the longitudinal reinforcement.

As shown in Fig. 14(b), at the wing wall face (location B), the strains of the longitudinal reinforcement in the left beam of specimen Z1 are less than those at its critical section, as illustrated in Fig. 14(a), which is in accordance with the theoretical distribution of the flexural moment in the beam. The strengthened specimen Z1-W reached the yield strain, which was much greater than the values at position A in Fig. 14(a). The critical section of the beam is shifted outwards from the column face of location A to the wing wall face of location B, where a plastic hinge is formed.

As shown in Fig. 14(c), at the critical section of the right beam, the strains of the longitudinal reinforcement are significantly increased by strengthening even though the wing walls were installed on the other side, indicating that the plastic hinge was more fully developed at that location.

#### Strain of anchors connecting the beam and wing walls

The strains of the anchor bars at the peak of every loading cycle are shown in Fig. 15. Under the negative loading direction, as shown in Fig. 6, the anchor bars in the lower wing wall are compressed, and those in the upper wing wall are tensile. The stresses of the anchors close to the column are small, and those far away are large, which indicates that the T-shaped section composed of the post-installed wing wall and the existing column worked together well and with stood the flexural moments, and the strain distribution at the sections agreed with the plane-section assumption.

The change trends of the values of the anchors in the wing wall on the same side are approximately the same.The values became larger with increasing distance from the column and increased with increasing loading displacement.Until the loading was stopped, the anchors did not yield.These phenomena indicated that the wing walls played a significant role in strengthening the joint, and the wing walls were not completely damaged.The strengthening efficiency is experimentally validated for interior joints by installing wing walls.

## Design method for strengthening interior joints via the wing wall installation method

**Fig. 16:**
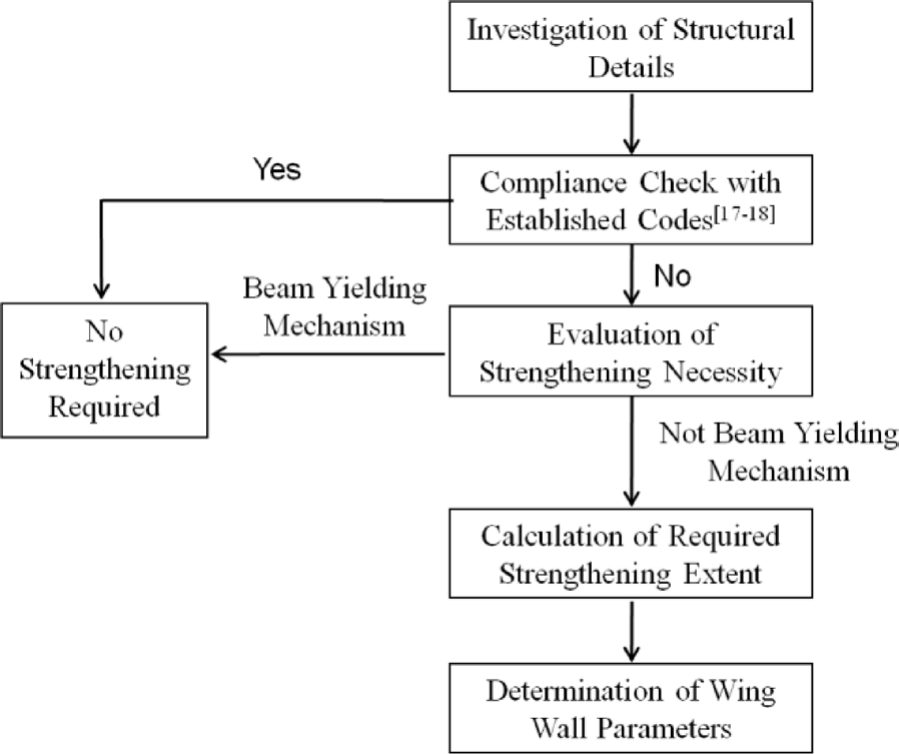
Design chart for strengthening design.

The test results validated the feasibility and effectiveness of strengthening interior beam‒column joints via the wing wall installation method. In the design for practical engineering strengthening, it is essential to first assess whether the joints of the existing structure require strengthening. If strengthening is deemed necessary, the extent of reinforcement must be determined; specifically, the design parameters of the wing wall should be established. The strengthening design chart can be illustrated as Fig. 16.

### Necessity determination for strengthening existing interior beam‒column joints

Structural details of the interior beam-column, such as dimensions, concrete strength, and reinforcement arrangement, must be thoroughly investigated and compared with the specifications of the relevant design codes^36–39^. If the existing details do not comply with these specifications, for instance, if stirrups are not provided in the joint region, as observed in the target building of this study, a strength evaluation shall be conducted as follows.

**Fig. 17:**
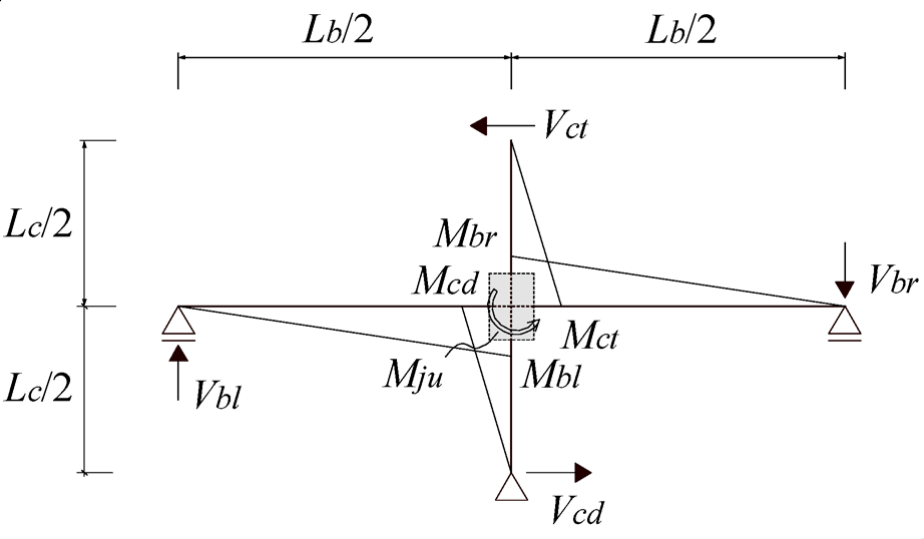
Shear and moment diagram of the interior beam‒column joint under seismic action.

Based on the calculation and comparison of the strengths of the beams, columns, and joints of the existing frame structure, a method for determining the necessity of strengthening the interior beam-column joints of the existing structure is proposed. The distributions of the flexural moment and shear force to an interior joint and its connected beams and columns under seismic action are shown in Fig. 17. By assuming that the beam length is *l*_*b*_, the half-length of the beams, i.e., the distance from the centre of the joint to the inflection point of the beam, is *l*_*b*_/2.The height of the columns is *l*_*c*_, and the half-height of the columns is *l*_*c*_/2.

Based on the parameters of the dimensions, reinforcement, and material strengths, the flexural strengths of the left beam *M*_*bu, l*_, the right beam *M*_*bu, r*_, the upper column *M*_*cu, t*_, and the lower column *M*_*cu, d*_ can be calculated according to the formulas given in the design code for concrete structures^41^.The nodal moment at the flexural strength of the critical section of the left and right beams, _j_*M*_*bu, l*_and _j_*M*_*bu, r*_,can be calculated by Eqs. (2) and (3), respectively. The nodal moment at the flexural strength of the critical section of the upper and lower columns,_j_*M*_*cu, t*_ and _j_*M*_*cu, d*_,can be calculated according to Eqs. (4) and (5), respectively.

2 $${}_{j}{M_{bu,l}}={M_{bu,l}} \cdot \frac{{{l_b}/2}}{{{l_b}/2 - {h_c}/2}}$$

3 $${}_{j}{M_{bu,r}}={M_{bu,r}} \cdot \frac{{{l_b}/2}}{{{l_b}/2 - {h_c}/2}}$$

4 $${}_{j}{M_{cu,t}}={M_{cu,t}} \cdot \frac{{{l_c}/2}}{{{l_c}/2 - {h_b}/2}}$$

5 $${}_{j}{M_{cu,d}}={M_{cu,d}} \cdot \frac{{{l_c}/2}}{{{l_c}/2 - {h_b}/2}}$$

Existing design codes^36,37^ provide formulas for calculating the strengths of beam-column joints. However, these formulas are applicable only to structures that comply with code specifications, and are not suitable for non-conforming structures—such as the target building in this study, where no stirrups are placed in the joint region. Shiohara proposed an innovative flexural resistance model for beam-column joints^42^ and introduced a method for calculating the flexural strength *M*_ju_^43^. This method takes into account structural details such as the amount of stirrups in the joint, thereby enabling accurate strength evaluation even in cases where no stirrups are present.

According to the SHIOHARA model^43^, the equations for calculating the strength reduction factor *β*_*j*_, which is defined as the ratio of the flexural strength of the joint to the nodal moment at flexural hinging at the adjacent beam end, have been provided in the literature^43^ for interior, exterior and corner joints. Because this study focuses on interior joints, the reduction factor for interior joints is given in Eq. (6). Consequently, the ultimate strength at joint hinging failure is given in Eq. (7).

6 $${\beta _j}={\xi _r}\left\{ {{\mathrm{1}} - \frac{{{A_t}{f_y}}}{{{b_j}{D_b}{F_c}}}+\frac{1}{{\mathrm{2}}}\left( {\frac{{{}_{j}{M_{cu,t}}+{}_{j}{M_{cu,d}}}}{{{}_{j}{M_{bu,l}}+{}_{j}{M_{bu,r}}}} - 1} \right)+\frac{1}{{\mathrm{4}}}\left( {\frac{{{A_j}{f_{jy}}}}{{A{}_{t}{f_y}}}} \right)} \right\}$$

7 $${M_{ju}}={\beta _j} \cdot \left( {{}_{j}{M_{bu,l}}+{}_{j}{M_{bu,r}}} \right)$$

Here, *ξ*_*r*_ is a reduction factor depending on *ξ* in Table 3; *ξ*is the aspect ratio, i.e., *D*_*b*_/*D*_*c*_; *D*_*b*_ is the depth of the beam;*D*_*c*_ is the depth of the column; *A*_*t*_ is the sectional area of the effective tensile reinforcement in the beam cross-section;*f*_*y*_ is the yield stress of longitudinal reinforcement steel in the beam; *b*_*j*_ is the effective width of the joint, which is the width of the column or the beam when their widths are the same; *F*_c_ is the compressive strength of the concrete; *A*_*j*_ is the gross sectional area of the shear reinforcement in the joint crossing the vertical plane; and *f*_*jy*_ is the yield stress of the joint shear reinforcement steel.

**Table 3:** Reduction factor as a function of the aspect ratio^43^.

ξ	0.7	0.8	0.9	1.0	1.2	1.4	1.6	1.8
*ξ*_*r*_	0.970	0.988	0.997	1.000	0.992	0.973	0.949	0.925

The ultimate strength *M*_fu_ of the partial frame composed of the non-strengthened interior joint of the connected beams and columns is determined by the minimum values of _j_*M*_*bu, l*_ + _j_*M*_*bu, r*_, _j_*M*_*cu, t*_ + _j_*M*_*cu, d*_ and *M*_*ju*_, as illustrated in Eq. (8). By comparison, the failure mode of the frame can be determined:

(1) if the quantity _j_*M*_*bu, l*_ + _j_*M*_*bu, r*_ is minimized, the corresponding failure mode is expected to be beam hinging. This mechanism is considered ideal, as it aligns with the design objectives of codes such as ASCE^39^, and therefore does not require strengthening.

(2) if _j_*M*_*cu, t*_ + _j_*M*_*cu, d*_ is the minimum, the failure mode should be column hinging, which needs to be strengthened; and (3) if *M*_*ju*_ is the minimum, the failure mode of the frame should be joint hinging, which does not satisfy the seismic design concept of"strong joints and weak members”, and it is necessary to strengthen the joints.

8 $${M_{fu}}=\hbox{min} [({}_{j}{M_{cu,t}}+{}_{j}{M_{cu,d}}),({}_{j}{M_{bu,l}}+{}_{j}{M_{bu,r}}),{M_{ju}}]$$

### Calculation of the required strengthening amount

For structures that need strengthening for joints according to the judgement method presented in Chap. 4.1, the minimum strengthening demand *M*_*need*_ can be calculated according to Eq. (9) by taking the objective that the joint hinging strength *M*_*ju*_ is equal to that of beam hinging (_j_*M*_*bu, l*_ + _j_*M*_*bu, r*_).

9 $${M_{need}} \geqslant \left( {{}_{j}{M_{bu,l}}+{}_{j}{M_{bu,r}}} \right) - {M_{ju}}$$

### Determination of the wing wall parameters

#### Forces between beams and the wing walls

**Fig. 18:**
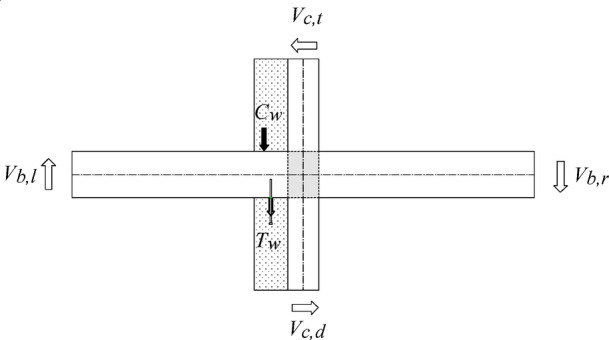
Actions from the wing walls to the existing beam.

From the stresses of the beam anchors recorded during the test, as illustrated in Fig. 15, it can be found that the compressed wing wall exerts a compressive force *C*_w_ on the beam and that the tensile wing wall produces a pull force *T*_w_ on the beam through the anchors, as shown in Fig. 18.

#### Mechanical model for strengthening interior joints by wing walls

**Fig. 19:**
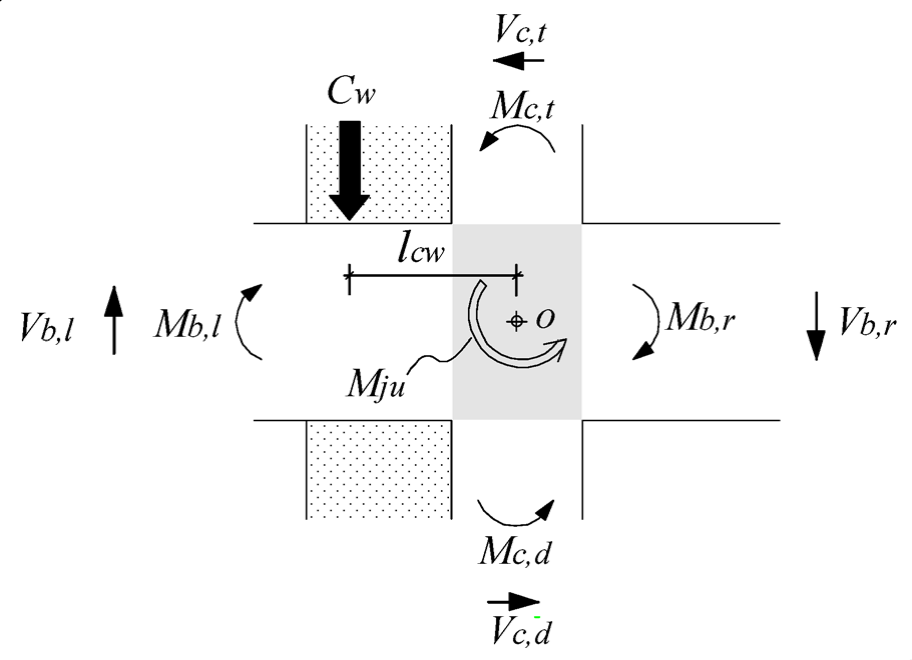
Forces around the strengthened interior joint under seismic action.

The compressive force *C*_*w*_ is mainly contributed by the concrete of the wing wall, and the pull force *T*_*w*_ is produced by the anchor bars connecting the wing wall and the beam. In the authors’ previous study^33^, a strengthening model in which the accurate joint hinging strength can be calculated considering the contributions of both the compressive and tensile forces was developed. However, the calculations are too complex and need to be simplified for practical design. Compared with the compressive force *C*_*w*,_ which is mainly produced by the concrete of the wing wall, the tensile force *T*_*w*_, which is produced by the anchor bars, is significantly less. If *T*_*w*_ is neglected, the hinging strength of the strengthened joint will be underestimated. However, a strengthening design will lead to improved safety. By neglecting *T*_*w*_, the force distribution around the joint region is shown in Fig. 19.The strengthening demand *M*_*need*_ is provided by the moment produced by the force *C*_w_ to the joint centre of *o*.By assuming that the distance from the action point of the force to the joint centre is *l*_*cw*_, the moment produced by *C*_*w*_ should be *C*_*w*_·*l*_*cw*_.This moment direction is the same as the joint hinging strength *M*_*ju*_. Hence, the pull force *C*_w_ improves the joint strength by *C*_*w*_·*l*_*cw*_.If the improvement is greater than the strengthening demand *M*_*need*_, i.e., Eq. (10) is satisfied, the strengthening objective can be successfully achieved.

10 $${C_w} \cdot {l_{cw}}>{M_{need}}$$

#### Methods for determining the wing wall parameters

**Fig. 20:**
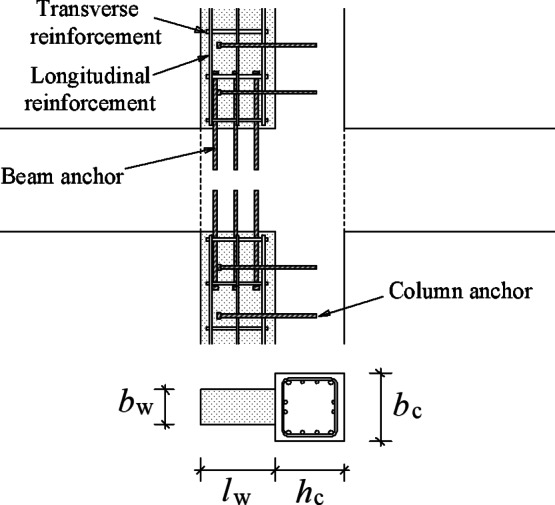
Main parameters of the wing walls.

As shown in Fig. 20, the parameters of the wing wall to be determined for the strengthening design include the amount of column anchors, beam anchors, longitudinal reinforcement and transverse reinforcement; the concrete strength *f*_*c*_; the wall thickness *b*_*w*_; and the wall length *l*_*w*_.The amount of column and beam anchors is determined in accordance with the detailing requirements for anchors in design codes, and the gross sectional area of the longitudinal and transverse reinforcements in the wing wall is greater than that of the beam and column anchor bars, respectively, to ensure that the post-installed wing wall has sufficient strength.The concrete strength *F*_*c*_ is set to be greater than that of the existing structure.The wing wall thickness *b*_*w*_ is taken to be an integer less than that of column *b*_*c*_, as shown in Fig. 20. Thus, the wing wall length is the only parameter that needs to be determined.

**Fig. 21:**
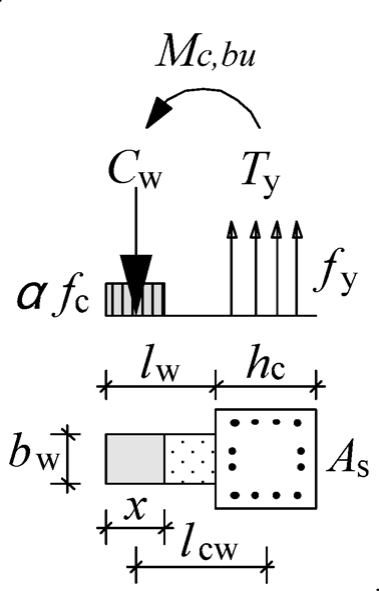
Forces at the T-shaped column section at the full plastic stage.

The objective of strengthening is to ensure that the structure forms a beam-end hinging mechanism. By installing wing walls, the cross section of the column changes from square to T-shaped, as shown in Fig. 21. Under the flexural moment *M*_*c, bu*_, at the critical section of the upper column, the longitudinal reinforcement in the column is tensile, and the concrete on the outer edge of the wing wall is under pressure. When the T-shaped section reaches the fully plastic state, the pull force *C*_*w*_ from the wing wall can be calculated according to the force equilibrium by Eqs. (11) and (12), i.e., the compressive force in the concrete is equal to the resultant force of the tensile stresses in the longitudinal reinforcement.

11 $${C_{\mathrm{w}}}={T_y}+N$$

12 $${T_y}={f_y} \cdot {A_s}$$

where *T*_*y*_ is the yield force of the longitudinal reinforcement in the column, *N* is the axial force to the column, *f*_*y*_ is the yield stress of the reinforcement, and *A*_*s*_ is the gross area of the longitudinal reinforcement in the column.

The relative depth of the concrete compression zone *x* can be obtained from Eq. (13).

13 $$x=\frac{{{C_w}}}{{\alpha {f_c} \cdot {b_w}}}=\frac{{{f_y} \cdot {A_s}+N}}{{\alpha {f_c} \cdot {b_w}}}$$

where *α* is the reduction factor of the concrete strength at the equivalent rectangular stress block.

The distance *l*_*cw*_ from *C*_*w*_ to the joint centre can be calculated by Eq. (14).

14 $${l_{cw}}=\left( {\frac{{{h_c}}}{2}+{l_w}} \right) - \frac{x}{2}=\left( {\frac{{{h_c}}}{2}+{l_w}} \right) - \frac{{{f_y} \cdot {A_s}+N}}{{2\alpha {f_c} \cdot {b_w}}}$$

By associating Eq. (14) with Eq. (9)~(12), Eq. (15) can be obtained. According to Eq. (15), the wing wall length *l*_*w*_ should satisfy Eq. (16).

15 $${f_y} \cdot {A_s} \cdot \left[ {\left( {\frac{{{h_c}}}{2}+{l_w}} \right) - \frac{{{f_y} \cdot {A_s}+N}}{{2\alpha {f_c} \cdot {b_w}}}} \right]>\left( {{}_{j}{M_{bu,l}}+{}_{j}{M_{bu,r}}} \right)$$

16 $${l_w}>\frac{{\left( {{}_{j}{M_{bu,l}}+{}_{j}{M_{bu,r}}} \right){\mathrm{-}}{M_{ju}}}}{{{f_y} \cdot {A_s}}}+\frac{{{f_y} \cdot {A_s}+N}}{{2\alpha {f_c} \cdot {b_w}}} - \frac{{{h_c}}}{2}$$

In summary, all the parameters of the wing wall are determined: the number of anchors in the columns and beams, the amount of longitudinal and transverse reinforcement of the wing wall, the concrete strength, and the thickness and the length of the wing wall.The wing wall can be designed based on the proposed theoretical analyses and calculations.

### Rationality confirmation of the wing wall design method

To verify the correctness of the proposed strengthening theory, the test results are evaluated based on the above design method. A comparison of the calculated and test results is shown in Table 3.

For the non-strengthened specimen Z1, the joint hinging strength *M*_*ju*_ is the smallest, and according to Eq. (8), the evaluated strength *M*_*fu*_ of the structure is 89.08kN.m. It is determined that the failure mode of the structure is joint hinging, and the structure needs to be strengthened.The maximum strength recorded in the test was 81.13 kN.m, and the failure mode was also joint hinging failure.Hence, the results from the theoretical evaluation are in agreement with the test results.

For the strengthened specimen Z1-W, the nodal moment is 214.13 kN.m at the flexural strength of the critical section of the T-shaped column after installing the wing wall. The nodal moment at the flexural strength of the critical section of the beam, i.e., when the beam is hinged at the wing wall face, is 122.41 kN.m. Based on the proposed strengthening mechanical model, the joint hinging strength *M*_*ju, r*_ after installing the wing walls can be calculated by Eq. (16).

17 $${M_{ju,r}}={M_{ju}}+{C_w} \cdot {l_{cw}}$$

By associating Eq. (17) with Equations (11) to (14), the strength of the strengthened joint is calculated to be 176.52 kN.m. The joint strength is improved by 87.44 kN·m due to the compressive force *C*_w_, as expressed in Eq. (17). If the tensile force *T*_w_ from the beam anchors, according to the previous accurate method^33^ and illustrated in Fig. 18, is also considered, the calculated joint strength would further increase by 24.22 kN·m, based on the yield stress of the anchors. However, this tensile contribution accounts for only 27.7% of the compressive contribution provided by *C*_w_. Therefore, neglecting the tensile contribution of the wing wall anchors represents a conservative simplification that favors safer design, albeit with a minor trade-off in quantitative accuracy.

Comparing the calculated strengths of the beam, column and joint of the strengthened specimen, it can be seen that the strength of the beam is the lowest. Hence, the strength of the specimen, *M*_*fu, r*_, is controlled by the beam (122.41 kN.m), and the failure mode of the structure is beam hinging, which approximately agrees with the test result of 127.17 kN.m.

**Table 3:** Comparisons between results from the proposed evaluation method and tests (kN.m).

Specimen	Column strength _j_M_cu, t_ + _j_M_cu, d_	Beam strength _j_M_bu, l_ + _j_M_bu, *r*_	Joint strength M_ju_	Evaluated results M_fu_	Tested results M_fu_
Z1	101.79	102.46	*89.08*	89.08, Joint hinging	81.13, Joint hinging
Z1-W	214.13	*122.41*	176.52	122.41, Beam hinging	127.17, Beam hinging

## Conclusions

A collapsed building due to joint failure in an earthquake was taken as the prototype in this study, and by modelling its interior beam‒column joints, two partial frame specimens, including the interior joint, were manufactured, one of which was seismically strengthened by the RC wing wall installation method. Pseudostatic testing was conducted on the specimens. From the test results, the brittle damage characteristics of the interior joint whose reinforcement details did not meet the structural requirements were confirmed, and the strengthening effectiveness of the wing wall installation method was analyzed.Taking the beam hinging mechanism as the strengthening objective, a method for determining the strength of interior beam‒column joints in existing frame structures was established. Based on a simplified mechanical model for strengthening interior joints with wing walls, a quantitative design method for wing walls was proposed, which provides a theoretical basis for practical strengthening design. The main conclusions are as follows:

1. The damage to the non-strengthened specimen was concentrated in the joint region, reproducing the failure state of the joints in the collapsed prototype building. After the maximum strength, the strength decreased significantly, showing obvious brittle failure characteristics.
2. The damage to the strengthened specimen by installing wing walls was concentrated at the critical sections of the beams, and the strength, ductility, and energy dissipation capacity were significantly improved.The failure mode of the structure changed to beam-ending yielding failure. The strengthening effectiveness of the wing wall installation method on interior beam‒column joints that do not satisfy the detailing requirements was validated.The strengthening objectives of protecting the joints and forming abeam hinging mechanism were achieved.
3. Based on the calculation and comparison of the strengths of the beam, column and joint, a method for determining the strength of the interior beam‒column joints was established.A simplified mechanical model for strengthening the interior joints with wing walls was proposed, and a method for determining the parameters of the wing wall was derived.
4. Based on the proposed design method for strengthening interior points via the wing wall installation method, the strengths and failure modes of the tested specimens were theoretically evaluated. The evaluated results approximately agreed with the test results, illustrating the rationality of the proposed strengthening theories.

In the present study, the experimental investigation was conducted on a limited number of interior beam-column joint specimens. To further validate and generalize the strengthening effectiveness, future research should include a wider range of joint configurations with varying geometric and material parameters. The influence of orthogonal beams and slabs on the performance of the strengthened joints also requires explicit examination. Additionally, sensitivity analyses, particularly regarding wall dimensions and material properties, are recommended to support the broader application of the proposed method.

## Data Availability

The data sets generated during the current study are available from the corresponding author on reasonable request.
